# Association between nutritional literacy and cancer anorexia among gastrointestinal cancer patients: a moderated mediation analysis on the roles of self-efficacy and family support

**DOI:** 10.3389/fonc.2026.1867483

**Published:** 2026-07-09

**Authors:** Peiyao Zhu, Zhen Li, Hongmei Zhang, Hengyu Hu, Xin Chen, Jingwen Cao, Yan Guo

**Affiliations:** 1 School of Nursing and Health, Henan University, Kaifeng, Henan, China; 2 Breast Surgery, Henan Provincial People’s Hospital, Zhengzhou, Henan, China; 3 Department of Nursing, Henan Provincial People's Hospital, Zhengzhou, Henan, China; 4 Department of Geriatric Medicine, Henan Provincial People's Hospital, Zhengzhou, Henan, China; 5 School of Nursing, Henan Medical University, Xinxiang, Henan, China; 6 Neurological Intensive Care Unit, Henan Provincial People's Hospital, Zhengzhou, Henan, China; 7 Cancer Center, Henan Provincial People's Hospital, Zhengzhou, Henan, China

**Keywords:** cancer anorexia, chemotherapy, family support, gastrointestinal cancer, moderated mediation model, nutrition literacy, self-efficacy

## Abstract

**Objectives:**

To test the relationship between nutritional literacy, self-efficacy, family support and cancer anorexic behavior among patients with gastrointestinal cancer undergoing chemotherapy. And examine the moderating role of family support on the relationship between nutritional literacy and self-efficacy.

**Methods:**

Using convenience sampling, 248 patients with gastrointestinal cancer undergoing chemotherapy were recruited from the Oncology Center of a Grade A Level 3 general hospital in Henan Province, China, between June and November 2025. Data analysis was performed by SPSS 27.0, while PROCESS macro v5.0 was used to test the moderated mediation model.

**Results:**

Nutritional literacy mediated by self-efficacy was positively associated with anorexic behavior score (*β* = 0.334, *t* = 6.156, *P*<0.001). Family support played a positive moderating role in the relationship between nutritional literacy and self-efficacy (*β* = 0.051, *t* = 4.704, *P*<0.001). When family support was high, nutritional literacy had a stronger association with self-efficacy.

**Conclusions:**

This study found that nutritional literacy among patients with gastrointestinal cancer is directly associated with cancer anorexic behavior and indirectly associated with it through self-efficacy. Furthermore, family support moderates the relationship between nutritional literacy and self-efficacy. Moving forward, interventions should be implemented on three fronts—improving patients’ nutritional literacy, strengthening their sense of self-efficacy, and enhancing family support systems—to alleviate cancer-related anorexia, improve nutritional status, and enhance quality of life.

## 1 Introduction

In 2023, the global incidence of cancer reached 18.5 million cases, projected to rise to 30.5 million by 2025, making it a major public health challenge threatening human health (1). Among these, gastrointestinal cancer accounts for over one-quarter of all cancer cases worldwide, constituting a significant component of the global cancer burden (2). Chemotherapy is one of the most commonly used and effective therapeutic modalities for gastrointestinal cancer, which can significantly improve overall survival rates and prognosis in the majority of patients (3). However, as a long-term treatment involving multiple cycles, chemotherapy inevitably damages normal cells while targeting cancer cells, leading to various side effects in patients (4). Among these, cancer anorexia is one of the most common and significant side effects, affecting over 80% of patients (5). Cancer anorexia (CA) is defined as a decrease or loss of appetite in cancer patients, with or without weight loss, which may occur throughout the course of the disease. Its occurrence is primarily associated with chemotherapy-induced taste alterations and gastrointestinal toxicity caused by chemotherapeutic agents (6, 7). Compared to other cancers, patients with gastrointestinal cancer experience higher rates of anorexia and more persistent symptoms. This is due to the primary cancer site directly impairing digestive and absorptive functions, coupled with targeted damage to the gastrointestinal mucosa caused by chemotherapy drugs. Anorexia leads to inadequate nutritional intake, compromising immunity and treatment tolerance. In severe cases, it may cause cachexia, significantly reducing chemotherapy compliance and quality of life while shortening survival time. Therefore, identifying the risk factors associated with the development of cancer-related anorexia in patients with gastrointestinal cancer and elucidating its underlying mechanisms is of great significance for improving patient prognosis and overall quality of life.

Nutrition literacy refers to an individual’s ability to obtain, understand, and apply nutrition information to maintain and promote their own health. It is one of the specific domains reflecting overall health literacy (8). As a vital component in promoting and maintaining healthy eating habits, nutrition literacy plays a crucial role in determining patients’ dietary behaviors (9). Previous studies have confirmed a strong association between individual nutrition literacy and dietary behaviors (10). Specifically, individuals with higher nutrition literacy exhibit more positive attitudes toward diet and demonstrate greater capacity to select and implement dietary practices aligned with dietary guidelines. They are better equipped to promptly curb or mitigate the onset and progression of unhealthy eating behaviors. For cancer patients, a lack of nutrition-related knowledge has become a significant barrier to maintaining a healthy diet. Research indicates that cancer patients with low nutritional literacy fail to recognize the adverse effects of malnutrition on subsequent treatment and recovery (11). During the later stages of chemotherapy, they often neglect their anorexic behaviors, lacking the awareness to self-adjust or actively seek professional help, resulting in a relatively poorer willingness to eat. Based on this, this study proposes Hypothesis 1: The nutritional literacy of gastrointestinal cancer patients undergoing chemotherapy may predict their anorexic behaviors.

With the advancement of social psychology, the influence of positive psychological resources on promoting healthy behaviors has garnered widespread attention. Among these, self-efficacy is one of the areas receiving greater attention. Self-efficacy is an individual’s belief in their ability to achieve a predetermined goal and to organize and execute a series of actions (12). According to the knowledge-attitude-practice (KAP) model, changes in individual health-related behaviors follow a progressive path of “knowledge acquisition-belief formation-behavioral implementation” (13). Knowledge serves as the foundation for behavioral change, while attitude acts as the key mediator linking knowledge and behavior. Nutritional literacy, as patients’ ability to access, understand and apply nutritional information to maintain and promote their own health, constitutes the knowledge foundation for coping with cancer anorexia. It covers not only functional nutritional knowledge, but also strategic capabilities to translate information into practice (14). These knowledge resources can boost patients’ confidence in overcoming anorexia, encourage them to take proactive steps to manage their diet—including adjusting food textures, optimizing mealtimes, and overcoming discomfort during meals—and ultimately reduce the severity of cancer-related anorexia and improve nutritional intake. Empirical research also indicates that patients’ nutritional literacy significantly and positively influences their self-efficacy (15). Patients with adequate nutritional literacy demonstrate greater confidence in adhering to dietary guidance, a higher likelihood of achieving healthy eating behaviors, and milder anorexic behaviors. Based on this, this study proposes Hypothesis 2: Self-efficacy may mediate the relationship between nutritional literacy and cancer anorexic behaviors.

Bandura’s theory of self-efficacy posits that self-efficacy can stem from an individual’s firsthand experiences of success, as well as from vicarious experiences and verbal persuasion (16). It is a dynamically constructed belief in one’s abilities within a specific context, and an individual’s assessment of their own capabilities depends on the interaction between task-specific knowledge and situational support resources (17). As an environmental factor, social support can enhance or diminish the effectiveness with which individual factors translate into self-efficacy. Patients who receive strong social support tend to demonstrate greater confidence in their ability to cope with their illness and are more proactive in managing their condition on their own (18). For patients with gastrointestinal cancer, nutritional literacy reflects their knowledge base and cognitive foundation regarding the specific challenge of coping with cancer-related anorexia. Family support, as a vital component of a patient’s social support system, constitutes a key source of situational support. Such situational support resources do not directly or uniformly influence all individuals’ self-efficacy; rather, their effectiveness depends on the individual’s existing level of task-specific knowledge. Family support can provide patients with emotional comfort, informational support, and practical assistance, thereby influencing the process by which nutritional literacy translates into self-efficacy. When patients possess a certain level of nutritional literacy, verbal encouragement, emotional support, and practical dietary care from family members can effectively strengthen their sense of self-efficacy. Conversely, if family support is insufficient, patients may struggle to put their nutritional knowledge into practice due to a lack of external assistance; even with a high level of nutritional literacy, their sense of self-efficacy may diminish as they face dietary challenges on their own. Based on this, this study proposes Hypothesis 3: Family support may moderate the relationship between nutritional literacy and self-efficacy.

**Figure 1:**
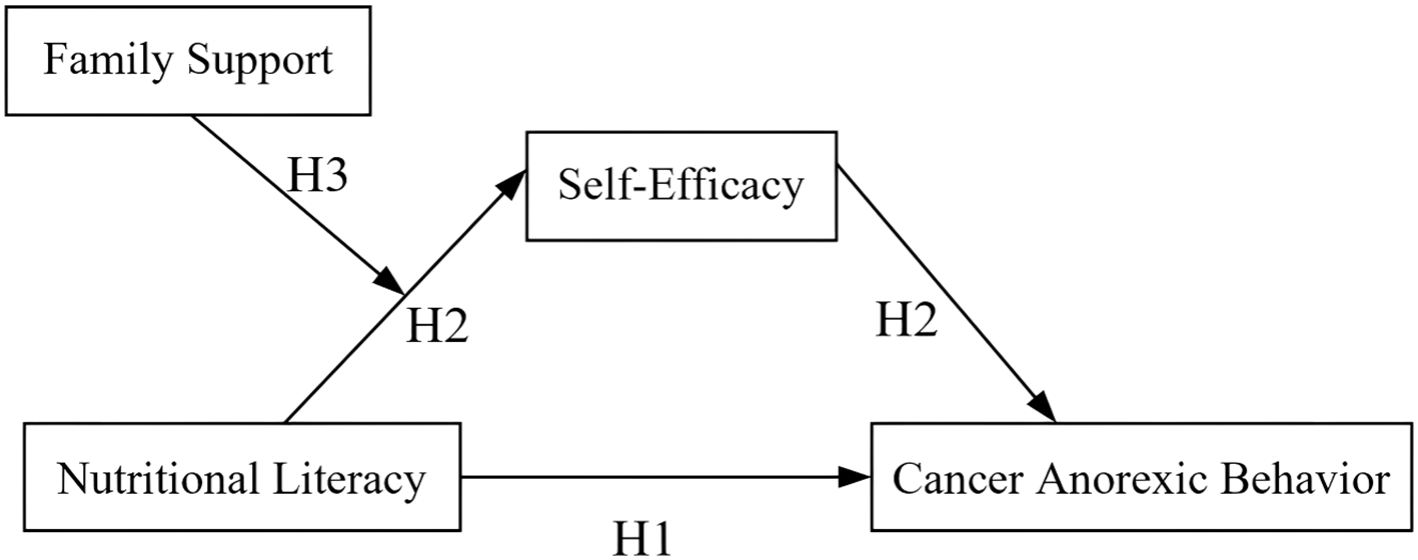
A moderated mediation model.

In summary, our study aims to investigate the relationship between nutritional literacy and anorexic behaviors among gastrointestinal cancer patients undergoing chemotherapy, as well as the mediating and moderating effects of self-efficacy and family support on this relationship. The findings are intended to provide theoretical guidance for further improving anorexic behavior in Patients with gastrointestinal cancer. The hypothetical framework is illustrated in **Figure 1**.

## 2 Methods

### 2.1 Study design and setting

This study is a cross-sectional design. Using convenience sampling, patients with gastrointestinal cancer undergoing chemotherapy at the Oncology Center of a tertiary hospital in Henan Province between June 2025 and November 2025 were selected as study subjects. The research adhered to the principles of the Declaration of Helsinki and was approved by the Ethics Committee of the researchers’ affiliated hospital [Ethics Approval No (2024). Lun Shen No (61).].

Inclusion Criteria (1): Age ≥ 18 years (2); Histopathologically confirmed gastrointestinal cancer (first diagnosis; no history of prior cancer treatment or recurrence), and currently undergoing chemotherapy (3); Clear diagnosis of the disease and understanding of disease progression (4); Adequate language comprehension and communication skills (5); Willingness to participate in the study and signing of an informed consent form. Exclusion Criteria (1): Individuals unable to swallow food orally (2); Individuals with anorexia nervosa (3); Individuals with psychiatric disorders or impaired consciousness (4); Individuals with concurrent diseases affecting other vital organs, those who are experiencing severe psychological trauma, or those whose attending physician has decided to temporarily suspend chemotherapy due to extreme weakness.

The sample size was determined based on a prior statistical power analysis conducted using the G*POWER 3.1.9.7 software. Use the decomposition method to break down the adjusted intermediate model into two regression submodels and calculate them separately. Submodel 1 was used to test the interaction effect (nutrition literacy × family support → self-efficacy) using linear multiple regression (F test). The effect size was set to a small-to-moderate effect size (f² = 0.085), which falls between Cohen’s small (f² = 0.02) and medium (f² = 0.15) thresholds and is appropriate for a conservative estimate of the interaction effect (19). α = 0.05, power (1-β) = 0.95, and three predictor variables (nutritional literacy, family support, and their interaction term). The calculation yields a sample size of 206 cases; taking into account a 10% non-response rate, the minimum required sample size is 229 cases. Submodel 2 was used to test the main effect (self-efficacy → anorexic behavior). Following standard practice, a moderate effect size (f^2^ = 0.15) was used as the benchmark, with α = 0.05. power (1-β) = 0.95, and two predictor variables (nutritional literacy, self-efficacy). The calculated sample size is 107 cases; considering a 10% non-response rate, the minimum required sample size is 134 cases. The larger of the two sub-sample sizes was selected as the final target sample size. The final effective sample size for this study was 248 cases, which met and exceeded the *a priori* sample size requirements.

### 2.2 Data collection

Using a questionnaire survey method, professionally trained researchers rigorously selected study subjects based on inclusion and exclusion criteria. Patients were informed of the study’s purpose through a standardized explanation and assured of confidentiality. After obtaining informed consent, the research team distributed QR codes for electronic questionnaires—previously created via the Wenjuangxing online platform—to patients in person. Staff assisted patients in scanning the QR codes to access the data entry page, where patients completed the forms independently. For patients unable to complete the questionnaire independently, the investigator will read the items aloud and complete them on their behalf, accompanying the patient throughout the process. To ensure the completeness of the questionnaire, it is configured to prevent submission if any items remain blank. A total of 260 questionnaires were distributed and collected. After excluding those completed in less than 3 minutes and those where over 90% of responses selected the same option, 248 valid questionnaires were recovered, yielding a valid response rate of 95.38%.

### 2.3 Measurements

#### 2.3.1 General information questionnaire

The general information questionnaire was developed by the research team following group discussions. A total of 16 entries, including general demographic information such as age, gender, educational attainment, marital status, BMI, etc. Disease-related information such as cancer location, cancer stage, number of chemotherapy cycles under way, etc.

#### 2.3.2 Independent variable: cancer survivor nutrition literacy assessment scale

This scale was developed in 2024 by Chinese scholars Xia et al. (20). Designed specifically to assess the nutritional literacy of cancer survivors, it takes into account the distinct nutritional requirements, absorption, and metabolic processes that distinguish cancer survivors from the general population. The scale comprises four dimensions: functional nutrition literacy (9 items), nutrition information access ability (4 items), nutrition knowledge application ability (5 items), and reasoning and calculation ability (3 items), totaling 21 items. The Functional Nutrition Literacy and Information Accessibility dimension employs a graded scoring system, with scores ranging from 1 to 7 in ascending order. Higher scores indicate greater proficiency in this dimension. For the Nutrition Knowledge Application and Reasoning/Calculation Ability dimensions, correct answers receive 1 point, while incorrect answers receive 0 points. A total score below 60 indicates inadequate nutritional literacy, with higher scores reflecting greater nutritional literacy. The overall Cronbach’s α coefficient for the scale is 0.830.

#### 2.3.3 Moderating variable: family adaptation, partnership, growth, affection, resolve index (APGAR)

This scale was developed by Smilkstein et al. (21) in 1978 and has since been widely used and validated across various populations and cultural contexts (22). It is primarily used to measure an individual’s perceived level of family support. Chinese scholars Lü et al. (23) conducted the Chinese adaptation in 1995. The scale comprises five items: adaptability, cooperativeness, growth orientation, emotionality, and intimacy. Using a 0–2 point rating scale, “always” scores 2 points, “sometimes” scores 1 point, and “rarely” scores 0 points. The sum of scores across the five items constitutes the total scale score. A total score of 7–10 indicates good family functioning, 4–6 indicates moderate family dysfunction, and 0–3 indicates severe family dysfunction. The overall Cronbach’s α coefficient for the scale is 0.894.

#### 2.3.4 Mediating variable: general self-efficacy scale (GSES)

This scale was developed by Schwarzer et al. (24) in 1995 and adapted for Chinese use by Zhang et al. (25) in the same year. It primarily measures individuals’ beliefs in their own ability to cope with various challenges and solve problems. The scale comprises 10 items, each scored from 1 to 4 points on a scale ranging from “completely incorrect” to “completely correct”. The minimum score is 10 points, and the maximum is 40 points. A higher score indicates stronger self-efficacy among patients. The overall Cronbach’s α coefficient for the scale is 0.870.

#### 2.3.5 Dependent variable: functional assessment of anorexia/cachexia therapy anorexia/cachexia subscale (FAACT-ACS)

This scale was developed by American scholars Ribaudo et al. (26) in 2000 and is currently the primary assessment tool widely used internationally to evaluate anorexia in cancer patients. Chinese scholars Zhou et al. (27) conducted its Chinese adaptation in 2017. The scale consists of 12 items using a 5-point Likert scale, where 0 indicates “strongly disagree” and 4 indicates “strongly agree”. The total score ranges from 0 to 48 points, with higher scores indicating a lower risk of anorexia. A total score ≤24 indicates the presence of anorexic behavior. The overall Cronbach’s α coefficient for the scale is 0.729.

### 2.4 Data analysis

Data analysis was performed using IBM SPSS Statistics for Windows version 27.0. Categorical variables were described using frequency and percentage. Continuous variables with skewed distributions were described using median and interquartile range. The Mann-Whitney U test and Kruskal-Wallis H test were employed to examine differences in cancer-related anorexia among distinct subgroups. The Harman single-factor method was employed to test for the presence of common method bias. Correlations between variables were examined using Spearman’s correlation coefficients, as this method is insensitive to non-normal distributions by focusing on variable ranks (28). Models 4 and 7 in the PROCESS program written by Hayes (29) test the mediating role of self-efficacy and the moderating role of family support, respectively. This approach was specifically selected because its bias-corrected bootstrap procedure (5,000 resamples, 95% confidence interval) is inherently robust to non-normal data, as it estimates effect distributions by resampling the original dataset rather than relying on asymptotic normality assumptions (30). Results are considered statistically significant when the 95% CI does not include 0 and *P* < 0.05.

## 3 Results

### 3.1 Common method bias test

Before data analysis, Harman’s single-factor test was performed to assess potential common method bias (CMB) (31). Ten factors had eigenvalues greater than 1, and the first factor accounted for 33.59% of the variance, which is below the recommended 40% threshold. These results indicate that serious common method bias was not present in this study.

### 3.2 Characteristics of the participants

The characteristics of the 248 patients with gastrointestinal cancer included in this study were summarized in **Table 1**. 68.5% were male, and 31.5% were female. 52.8% of patients were aged 60 years or older. Based on the FAACT-ACS scoring criteria, 23% patients scored >24, indicating no risk of anorexic behavior, while the remaining 77% of patients exhibited varying degrees of anorexic behaviors. Significant differences in cancer anorexic behavior scores were found across BMI, cancer location, cancer stage, number of chemotherapy cycles under way, drinking alcohol, and receipt of nutrition education or lectures (*P* < 0.05). Therefore, we adjusted for these factors as control variables in subsequent analyses.

**Table 1:** Comparison of different characteristics of participants (N = 248).

Variables	Group	*N (%)*	*M(P25, P75)*	*Z/H*	*P*
Gender	Male	170(68.5)	21.00 (17.00, 25.00)	-1.056^(a)^	0.291
	Female	78(31.5)	20.00 (17.00, 23.25)		
Age (years)	18-39	9(3.6)	20.00 (16.50, 25.50)	0.000^(b)^	1.000
	40-59	108(43.5)	20.50 (17.00, 24.00)		
	≥60	131(52.8)	20.00 (17.00, 24.00)		
Marital Status	Single/Divorced/Widowed	11(4.4)	20.00 (17.00, 25.00)	-0.013^(a)^	0.990
	Married	237(95.6)	20.00 (17.00, 24.00)		
Educational Level	Primary school and below	88(35.5)	20.00 (17.00, 24.00)	6.967^(b)^	0.073
	Junior high school	94(37.9)	21.00 (19.00, 26.00)		
	High school or technical secondary school	42(16.9)	21.00 (16.75, 24.00)		
	University (including junior college) or above	24(9.7)	17.00 (16.00, 23.00)		
Household Monthly Income Percapita (CNY)	<3 000	124(50.0)	20.00 (17.00, 24.00)	0.947^(b)^	0.623
	30 00-5 000	97(39.1)	20.00 (17.00, 25.00)		
	>5 000	27(10.9)	21.50 (17.00, 23.00)		
Main Carer	Parents	29(11.7)	20.00 (16.50, 24.00)	4.547^(b)^	0.208
	Spouse	124(50.0)	20.50 (17.00, 24.00)		
	Children	74(29.8)	20.00 (17.00, 23.00)		
	Relatives and friends	21(8.5)	22.00 (20.00, 26.50)		
BMI (kg/m²)	<18.5	108(43.5)	21.00 (18.25, 24.75)	10.420^(b)^	**0.005**
	18.5-23.9	127(51.2)	20.00 (16.00, 24.00)		
	24-27.9	13(5.2)	16.00 (15.25, 20.25)		
Number of Weekly Physical Exercise Sessions	<1	52(21.0)	19.00 (16.00, 21.00)	6.686^(b)^	0.083
	1-2	53(21.4)	20.00 (16.50, 24.00)		
	3-4	47(19.0)	21.00 (17.00, 24.00)		
	5-7	96(38.7)	21.50 (17.25, 25.00)		
Cancer location	Stomach	106(42.7)	20.00 (16.00, 23.25)	12.644^(b)^	**0.005**
	Colorectal	60(24.2)	22.00 (17.00, 25.00)		
	Esophagus	32(12.9)	17.00 (15.25, 25.00)		
	Liver**^#^**/Gallbladder/Pancreas	50(20.2)	21.50 (20.00, 24.00)		
Cancer Stage (TNM)	I	18(7.3)	21.00 (18.75, 22.25)	15.478^(b)^	**0.001**
	II	90(36.3)	22.00 (20.00, 25.00)		
	III	80(32.3)	20.00 (17.00, 23.75)		
	IV	60(24.2)	17.00 (15.25, 25.00)		
Treatment Methods	Surgery + Chemotherapy	101(40.7)	20.00 (17.50, 23.00)	3.743^(b)^	0.291
	Surgery + Chemotherapy + Radiation Therapy	30(12.1)	22.50 (16.75, 26.25)		
	Surgery + Chemotherapy + Targeted Therapy	51(20.6)	21.00 (17.00, 24.00)		
	Chemotherapy + Targeted Therapy	66(26.6)	20.00 (16.00, 25.00)		
Number of Chemotherapy Cycles Under Way	1	49(19.8)	23.00 (18.00, 26.00)	22.791^(b)^	**<0.001**
	2	71(28.6)	21.00 (19.00, 25.00)		
	3	50(20.2)	20.00 (16.00, 22.00)		
	≥4	78(31.5)	18.00 (16.00, 21.00)		
Combined Chronic Diseases	No	171(69.0)	20.00 (17.00, 24.00)	-1.342^(a)^	0.179
	Yes	77(31.0)	21.00 (18.00, 25.00)		
Drinking Alcohol	No	187(75.4)	21.00 (18.00, 25.00)	-2.639^(a)^	**0.008**
	Yes	61(24.6)	19.00 (16.00, 22.50)		
Smoking	No	189(76.2)	21.00 (17.00, 25.00)	-1.751^(a)^	0.080
	Yes	59(23.8)	20.00 (16.00, 23.00)		
Receipt of Nutrition Education or Lectures	No	186(75.0)	18.00 (16.00, 23.00)	-2.748^(a)^	**0.006**
	Yes	62(25.0)	21.00 (17.00, 24.25)		

**^#^**All liver cancer cases in this study were pathologically confirmed intrahepatic cholangiocarcinoma (ICC) and received chemotherapy. (a): Mann-Whitney U Test; (b): Kruskal-Wallis H Test. Bolded numbers indicate P < 0.05.

### 3.3 Scores and correlations among variables

Patients undergoing chemotherapy for gastrointestinal cancer scored 20.00 (17.00, 24.00) on anorexia behavior, 50.00 (39.00, 64.00) on nutritional literacy, 8.00 (7.00, 8.00) on family support, and 28.00 (25.00, 30.00) on self-efficacy. Relevant analysis indicates that there is a significant correlation between each pair of variables (**Table 2**).

**Table 2:** Correlation analysis results among variables.

Variables	Nutritional literacy	Family support	Self-efficacy	Anorexic behavior
Nutritional Literacy	1			
Family Support	0.419**	1		
Self-Efficacy	0.514**	0.332**	1	
Anorexic Behavior	0.646**	0.532**	0.570**	1

**P<0.05.

### 3.4 Mediating effect of self-efficacy on the relationship between nutritional literacy and cancer anorexic behavior

Mediator effect analysis was conducted using Model 4 from the PROCESS macro program. The independent variable was nutritional literacy, the mediating variable was self-efficacy, and the dependent variable was cancer anorexic behavior score. The control variables included BMI, cancer location, cancer stage, number of chemotherapy cycles under way, drinking alcohol, and receipt of nutrition education or lectures.

Results indicate that nutritional literacy is significantly and positively associated with patients’ self-efficacy (*β* = 0.142, *P* < 0.001). Self-efficacy is significantly and positively associated with anorexic behavior score (*β* = 0.334, *P* < 0.001). Nutritional literacy is significantly and positively associated with anorexic behavior score (*β* = 0.138, *P* < 0.001). Therefore, Hypothesis 1 is validated. The total effect of nutritional literacy on anorexic behavior is 0.186 (*95% CI*: 0.158-0.213, *P* < 0.001). The direct effect is 0.138 (*95% CI:* 0.108-0.168, *P* < 0.001), accounting for 74.19% of the total effect; the indirect effect is 0.048 (9*5% CI*: 0.034-0.062, *P* < 0.001), accounting for 25.81% of the total effect. Hypothesis 2 is thus validated (**Tables 3**, **4**).

**Table 3:** Testing the mediating model of self-efficacy.

Item	Self-efficacy		Anorexic behavior score		Anorexic behavior score	
	*β*	*t*	*β*	*t*	*β*	*t*
Nutritional Literacy	0.142	9.231	0.186	13.332	0.138	9.151
Self-Efficacy	–	–	–	–	0.334	6.156
*F*	13.440^***^		32.822^***^		37.870^***^	
*R^2^*	0.282		0.489		0.559	

^***^ *P*<0.001.

**Table 4:** Total effect, direct effect, and indirect effect.

Item	Effect	SE	LLCI	ULCI	Efficiency ratio (%)
Total Effect	0.186	0.014	0.158	0.213	100
Direct Effect	0.138	0.015	0.108	0.168	74.19
Indirect Effect	0.048	0.007	0.034	0.062	25.81

Therefore, self-efficacy partially mediates the relationship between nutritional literacy and anorexic behavior in patients undergoing chemotherapy for gastrointestinal cancer. Specifically, patients’ nutritional literacy is directly associated with their anorexic behavior and also indirectly associated with it by influencing their self-efficacy.

### 3.5 Moderated mediation effect of family support on self-effect in the relationship between nutrition literacy and cancer anorexic behavior

**Figure 2:**
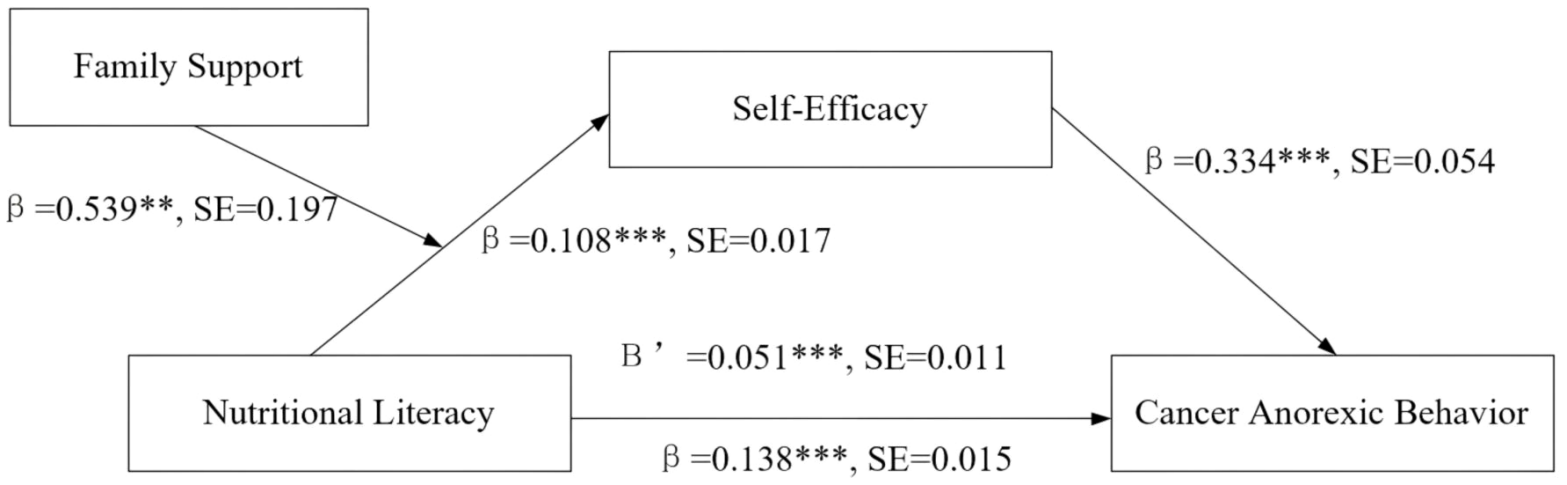
Moderating effect model of family support on the relationship between nutritional literacy and cancer anorexic behavior in patients with gastrointestinal cancer undergoing chemotherapy. B’, Nutritional Literacy*Family Support. **P<0.05; ***P<0.001.

Using Model 7 in the PROCESS macro program, we further examined the moderating effect of family support on the mediating pathway. Before conducting the moderated mediation analysis using PROCESS Model 7, the independent variable and the moderator were mean-centered to reduce multicollinearity and facilitate interpretation of the conditional effects. Results indicate that the interaction term between nutritional literacy and family support significantly predicted self-efficacy among patients undergoing chemotherapy for gastrointestinal cancer (*β* = 0.051, *P* < 0.001), suggesting that family support moderates the effect of nutritional literacy on self-efficacy. Thus, Hypothesis 3 is supported (**Table 5**; **Figure 2**).

**Table 5:** Testing the moderating role of family support in the mediating model.

Item	Self-efficacy			Anorexic behavior score		
	*β*	*SE*	*t*	*β*	*SE*	*t*
Constant	26.260	2.037	12.892***	17.033	2.308	7.380***
Nutritional Literacy	0.108	0.017	6.527***	0.138	0.015	9.151***
Family Support	0.539	0.197	2.735**	–	–	–
Self-Efficacy	–	–	–	0.334	0.054	6.156***
Nutritional Literacy × Family Support	0.051	0.011	4.704***	–	–	–
*F*	14.598***			37.870***		
*R^2^*	0.356			0.559		

**P<0.05; ***P<0.001.

**Figure 3:**
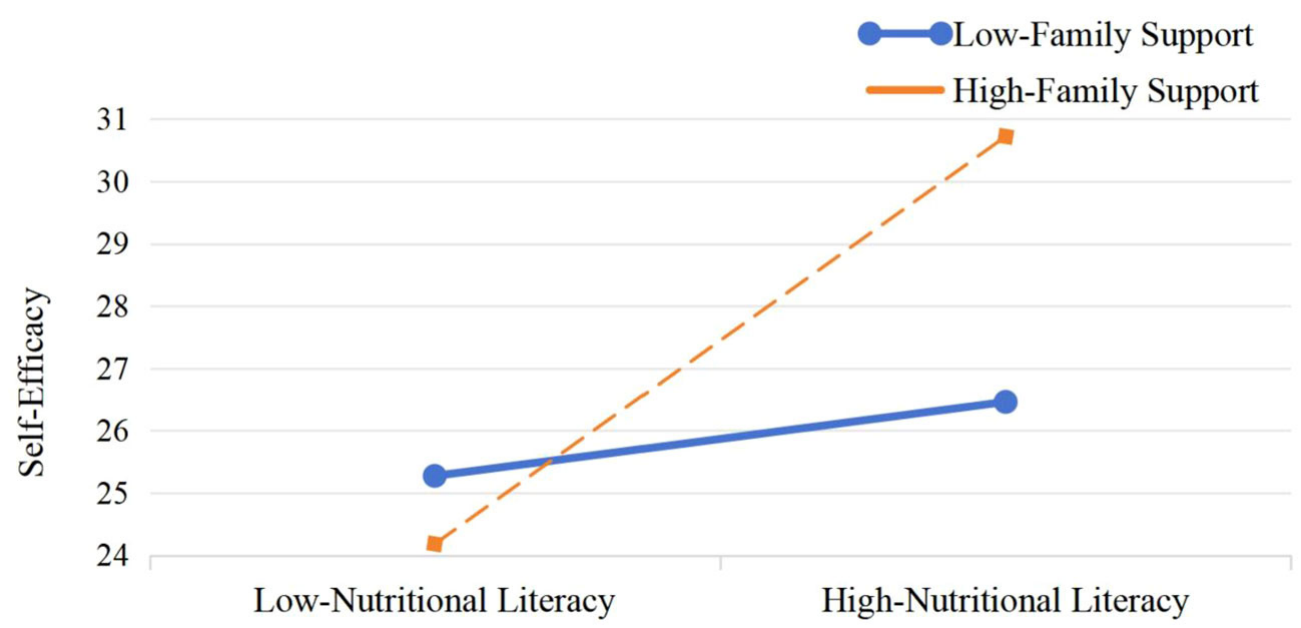
Moderating effect diagram of family support.

To explore the substantive nature of the interaction effects between nutritional literacy and family support, this study further conducted a simple slope analysis. The results indicate that, at low levels of family support (-1 SD), the predictive effect of nutritional literacy on self-efficacy is not significant (β = 0.033, 95% CI: -0.017-0.084); At the mean level of family support, nutritional literacy has a significant positive effect on self-efficacy (β = 0.108, 95% CI: 0.076-0.141); At high levels of family support (+1 SD), the effect of nutritional literacy on self-efficacy is further enhanced (β = 0.184, 95% CI: 0.144-0.223). The adjusted mediation coefficient was 0.017 (95% CI: 0.010-0.026); since the confidence interval does not include 0, this indicates that the adjusted mediation effect is significant (**Table 6**; **Figure 3**).

**Table 6:** Effects of nutritional literacy on self-efficacy at different levels of family support.

Family support	Effect	BootSE	BootLLCI	BootULCI
-1SD (-1.469)	0.033	0.026	-0.017	0.084
Mean (0)	0.108	0.017	0.076	0.141
+1SD (1.469)	0.184	0.020	0.144	0.223
Moderated Mediation Effect	0.017	0.004	0.010	0.026

## 4 Discussion

### 4.1 The association between nutritional literacy and anorexic behavior

The results of this study indicate that nutritional literacy among patients undergoing chemotherapy for gastrointestinal cancer shows a significant association with anorexic behavior, supporting Hypothesis 1. Specifically, the higher a patient’s level of nutritional literacy, the higher their anorexia score—indicating relatively milder anorexic symptoms—which is consistent with the findings of Malaysian researchers Siow YY et al. (32). Patients with higher levels of nutritional literacy are more likely to follow dietary guidelines provided by healthcare professionals. They have a clearer understanding of how their nutritional needs change during chemotherapy and can effectively alleviate loss of appetite by making appropriate adjustments to their diet, eating patterns, and mealtimes, thereby reducing the onset and progression of anorexia (33). These findings suggest that nutritional literacy may play an important role in the early identification and management of patients at risk of cancer-related anorexia. Therefore, in clinical practice, patients with low nutritional literacy should be promptly identified and screened. Appropriate assessment tools can be used to screen for nutritional literacy upon admission and during chemotherapy to identify patients who require targeted nutritional guidance. In recent years, researchers have developed a photo-based nutritional literacy assessment tool that is particularly suitable for individuals with low educational attainment or communication difficulties (34). Future efforts could focus on promoting its cross-cultural validation among cancer patients to enhance the convenience and accuracy of nutritional literacy screening.

### 4.2 The mediating role of self-efficacy between nutritional literacy and anorexic behavior

This study confirms that self-efficacy acts as a mediating mechanism between nutritional literacy and anorexic behavior, demonstrating both direct and indirect effects, which support Hypothesis 2. This finding is consistent with the Knowledge-Attitude-Practice (KAP) model, which posits that knowledge serves as the foundation for behavioral change, while beliefs act as the key mediating factor linking knowledge and behavior (13). On the one hand, nutritional literacy is a significant predictor of patients’ self-efficacy. When patients have a high level of nutritional literacy, they can accurately interpret food labels, understand the expert recommendations in dietary guidelines, and translate abstract nutritional knowledge into daily dietary decisions that help combat anorexic behaviors. This process continuously builds patients’ positive experiences of mastery over their illness, and these experiences of mastery are the most fundamental and stable source of self-efficacy (35). On the other hand, patients’ levels of self-efficacy significantly predict their anorexic behaviors. Individuals with high self-efficacy are more likely to persist in goal-oriented behaviors when faced with obstacles. When patients have a high sense of self-efficacy regarding dietary management, they are more likely to view anorexic behaviors as temporary, manageable physiological responses rather than signs of disease progression. This positive mindset reduces anxiety and fear during mealtimes, making them more willing to proactively adopt positive coping strategies, such as choosing high-energy-density liquid foods over solid foods when their appetite decreases, or adjusting their mealtimes during the peak of nausea following chemotherapy (36). Conversely, patients with low self-efficacy often attribute their anorexic symptoms to a lack of personal ability or the uncontrollable nature of the illness; when faced with difficulties eating, they are more likely to resort to avoidance behaviors. In summary, the mediating role of self-efficacy revealed in this study has potential clinical significance, suggesting that healthcare professionals should recognize the synergistic role of nutritional literacy and self-efficacy in managing anorexia in patients with gastrointestinal cancer, particularly the patients’ sense of self-efficacy. Clinicians should focus on identifying individuals with low self-efficacy and use methods such as nutrition education, role modeling, and emotional support (37) to enhance patients’ ability to self-manage anorexia and boost their confidence in treatment.

### 4.3 The moderating role of family support between nutritional literacy and self-efficacy

The results of the moderation analysis indicate that the interaction between nutritional literacy and family support significantly predicts self-efficacy among patients undergoing chemotherapy for gastrointestinal cancer, suggesting that family support moderates the first half of the mediating path. This supports Hypothesis 3. A simple slope analysis indicates that, under conditions of low family support, the predictive effect of nutritional literacy on self-efficacy is not significant. It should be noted that in the absence of family support, even patients with high nutritional literacy may struggle to develop a stable sense of self-efficacy due to a lack of effective social reinforcement. According to self-efficacy theory, a sense of self-efficacy is primarily formed through four pathways: experiences of control, vicarious experiences, verbal persuasion, and physiological and emotional states (16). In settings with low family support, multiple pathways are significantly constrained: patients lack assistance from family members, making it difficult for them to gain experience in successfully managing the complex dietary requirements during chemotherapy; the absence of positive feedback from close relationships weakens the effectiveness of verbal persuasion; and, compounded by the anxiety and helplessness of coping alone with loss of appetite, these negative emotional states further undermine their positive self-assessment of their own capabilities. These findings suggest that family support plays an important contextual role in shaping the effectiveness of nutritional literacy in enhancing patients’ self-efficacy. Patients with insufficient family support may be less able to translate nutritional knowledge into confidence in managing dietary challenges during chemotherapy. Therefore, improving patients’ nutritional literacy alone may not yield the desired results; it is essential to simultaneously strengthen the development of family support systems. Early identification of patients with limited family support may help clinicians provide more targeted psychological and nutritional guidance. Future studies are needed to develop feasible screening tools and intervention strategies that integrate both individual and family-level factors in this population. Studies have shown that family-centered empowerment interventions can significantly improve patients’ self-efficacy (38). It is recommended that clinicians adopt family-centered interventions, incorporating family members into the intervention process through family meetings and caregiver training. This approach ensures that improvements in nutritional literacy are reinforced and consolidated through positive social interactions, thereby maximizing the efficiency with which nutritional literacy is translated into self-efficacy.

## 5 Limitations and future research directions

This study has certain limitations. First, the research sample was drawn from a tertiary general hospital in Henan Province, resulting in limited geographical and hospital-level diversity. Future research should broaden the sample sources to include patients from different regions, hospitals of varying levels, and different cancer types to enhance the generalizability of the findings. Second, the cross-sectional design measured nutritional literacy, family support, self-efficacy and anorexic behavior at a single time point, limiting the ability to draw definitive causal inferences among variables. Future studies should employ longitudinal or cohort study designs, using repeated measurements at multiple time points to further clarify the dynamic interactions and causal relationships among variables.

## 6 Clinical implications

Despite these limitations, the moderated mediation model constructed in this study provides important theoretical foundations and practical guidance for developing future interventions aimed at improving anorexic behaviors in patients undergoing chemotherapy for gastrointestinal cancers. To our knowledge, this represents the first exploration in China of the relationship between nutritional literacy, self-efficacy, family support, and anorexic behaviors among patients with gastrointestinal cancers. Based on the findings of this study, clinical healthcare providers should not only recognize the direct impact of nutritional literacy on anorexic behavior but also emphasize the mediating and moderating roles of self-efficacy and family support in this process. By enhancing patients’ nutritional literacy, establishing appropriate family support systems, and boosting patients’ psychological motivation, collaborative efforts can effectively improve anorexic behavior.

## 7 Conclusions

The findings of this study reveal that (1): Nutritional literacy is significantly associated with anorexic behavior in patients undergoing chemotherapy for gastrointestinal cancers (2). Self-efficacy partially mediates the relationship between nutritional literacy and anorexic behavior in patients undergoing chemotherapy for gastrointestinal cancers (3). Family support moderates the first half of the mediation pathway: nutritional literacy → self-efficacy → anorexic behavior. These findings provide targets for clinical intervention, suggesting that healthcare professionals may improve anorexic behavior in gastrointestinal cancer patients by enhancing nutritional literacy, strengthening self-efficacy, and regulating family support levels.

## Data Availability

The raw data supporting the conclusions of this article will be made available by the authors, without undue reservation.
